# Poisoning by Carbolic Acid

**Published:** 1879-07

**Authors:** 


Poisoning by Carbolic Acid.—A case of acute poisoning with carbolic acid through an enema is recorded by Dr. Praetorius in the *Berliner Klinische Wochenschrift* April 14th, 1879. The patient, a delicate lady aged forty-five, had been suffering for several weeks from an obstinate attack of diarrhœa, which could not be stopped by the usual agents, and threatened to undermine the patient's strength. The author prescribed an enema of one per cent. solution of carbolic acid, of which a quarter of *litre* was mixed with one-third of a *litre* of warm water. Hardly had one-third of this enema been injected, when the patient began to complain of singing in the ears, giddiness, and weakness, and collapsed. The enema was of course immedietely suspended, and the patient told to void her bowels. This was done; but she remained in a collapsed state till the bowels had been washed out with warm water, when she gradually recovered; but it was not till two hours later that the disagreeable symptoms disappeared. The diarrhœa, however, had been stopped by the carbolic acid.—*Hospital Gazette.*

